# Unveiling the Biological Potential of Indigenous Oscillatoria spp. From Freshwater and Marine Ecosystems Through Advanced Characterization

**DOI:** 10.1002/fsn3.70868

**Published:** 2025-09-01

**Authors:** Jannatul Nayeem, Proma Dey, Sumit Kanti Dey, Helena Khatoon

**Affiliations:** ^1^ Department of Aquaculture, Faculty of Fisheries Chattogram Veterinary and Animal Sciences University Chattogram Bangladesh

**Keywords:** biochemical composition, cyanobacteria, growth curve, *Oscillatoria* sp., pigment, proximate composition

## Abstract

*Oscillatoria* species are considered versatile, eco‐friendly bio‐factories with significant potential for diverse sustainable innovations and applications. Characterizing *Oscillatoria* sp. is crucial for realizing their full potential as they exhibit differential growth patterns and produce various secondary metabolites. This study aims to systematically characterize and compare two freshwater and two marine *Oscillatoria* species. Thus, growth phases, nutritional composition, biochemical profiles (fatty acid and amino acid) and pigment contents of *Oscillatoria* species were analyzed. *Oscillatoria* species were cultured for growth curve determination and eventually mass cultured, harvested, and oven‐dried for the analyses. The growth curve of *Oscillatoria* spp. was assessed through chlorophyll‐a and optical density. Crude protein, lipid, and carbohydrate contents varied from 21.56% ± 0.09% to 56.97% ± 0.03%, 9.07% ± 0.07% to 17.13% ± 0.13%, and 7.49% ± 0.15% to 17.04% ± 0.08%, respectively. Mono‐unsaturated fatty acids were found higher in *Oscillatoria* spp. than saturated fatty acids. Non‐essential amino acids (61.12% ± 0.05% to 64.62% ± 0.03%) were found higher than essential amino acids (35.38% ± 0.02% to 38.84% ± 0.04%). Higher pigment contents were found in highly filamentous *Oscillatoria* spp. than in planktonic species. This comprehensive approach facilitates the targeted characterization and selection of *Oscillatoria* spp. with desirable traits, ensuring their effective and specific utilization. Optimizing large‐scale cultivation and efficient extraction of valuable metabolites from *Oscillatoria* sp. can significantly advance their potential benefits and integration into diverse commercial applications.

## Graphical abstract

This study approach enables the precise selection and screening of Oscillatoria spp., which is essential for identifying species with desirable properties, ensuring their effective utilization and maximizing their potential benefits across diverse commercial applications.

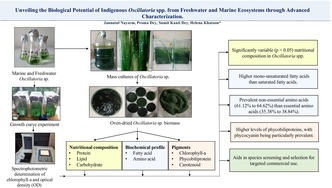

## 1 Introduction

Cyanobacteria are ancient gram‐negative prokaryotic autotrophs. They are renowned for their ability to perform oxygenic photosynthesis and generate substantial biomass across various environments, including non‐arable lands and wastewater sources. Their resilience and productivity make them a valuable bioresource for sustainable development (Singh, Kumar, et al. 2016). These microorganisms are prevalent in marine and freshwater ecosystems, particularly in tropical and subtropical waters, where they fix nitrogen or release organic carbon (Rajaneesh et al. 2020). Cyanobacteria may appear as single cells, clusters, or filaments. While cyanobacteria are typically microscopic, they become visible when they aggregate into colonies, such as in crusts or blooms (Catherine et al. 2013).

Bloom‐forming cyanobacterial genera are often undesirable in aquaculture as approximately 46 species from genera such as *Microcystis*, *Lyngbya*, *Anabaena*, *Cylindrospermopsis*, *Synechococcus*, and *Oscillatoria* produce toxins that cause toxic effects in aquatic systems (Aklakur et al. 2023). However, these same cyanobacterial genera are also notable for synthesizing a broad spectrum of bioactive compounds, including phenolic acids, flavonoids, tannins, and terpenoids, fatty acids with potent antimicrobial, anticancer, cytotoxic, immunomodulatory, and protease inhibitor effects (Bouyahya et al. 2024).

The rapid growth and high yield of cyanobacteria make them highly advantageous for large‐scale commercial cultivation. This enables the production of biofuels and other valuable products such as antioxidants, antimicrobials, biodegradable polymers, nutrient supplements, biofertilizers, and natural pigments (Zahra et al. 2020). The nutritional content in the dried cyanobacterial biomass generally comprises46% to 633% protein,4% to 222% lipids, and8% to 177% carbohydrates and vitamins (Loaiza et al. 2016).

*Oscillatoria* sp. has the potential to produce a variety of pigments, including chlorophyll, xanthophyll, astaxanthin, *β*‐carotene, and phycobiliprotein (Zuorro et al. 2021). The majority of the bioactive substances from *Oscillatoria* sp. are composed of fatty acids and amino acids that exhibit anti‐algal, antibacterial, antiviral, antiprotozoal, and antifungal properties (Contreras‐Ropero et al. 2022). Their secondary metabolites exhibit significant inhibitory effects against various genera of both gram‐positive (Mycobacterium, Staphylococcus, Bacillus, Listeria) and gram‐negative (Pseudomonas, Serratia, Escherichia, Aeromonas) bacteria (Rojas et al. 2020; Filatova et al. 2021).

Despite the potential of cyanobacteria, only 10% of the existing cyanobacterial species have been thoroughly characterized (Idenyi et al. 2021). Only a limited number of cyanobacterial species (Anabaena sp., Spirulina platensis, Nostoc sp., Galdieria sulphuraria, *Porphyridium cruentum* , and *Phormidium valderianum* ) are produced on an industrial scale (Jaeschke et al. 2021; Seghiri et al. 2021). Thus, there is a strong need for fundamental research to uncover and characterize new cyanobacterial species capable of producing high‐value products. Few studies have addressed the selective aspects of *Oscillatoria* sp. characterization. According to a recent review on the characterization of 111 cyanobacterial species (Passos et al. 2023), lipid and fatty acid content of freshwater *Oscillatoria* sp. was assessed for biodiesel production (Yadav et al. 2021) and fatty acid profiles for biomarkers (Sahu et al. 2013). The potential effect of temperature on freshwater *Oscillatoria* sp. biomass production and biochemical composition was also determined (Idenyi et al. 2021). There is limited data on marine *Oscillatoria* sp. and species‐specific biochemical variability, and there is no prior research that thoroughly characterizes and representatively compares both freshwater and marine *Oscillatoria* spp. Thus, this study aimed to characterize and compare freshwater and marine *Oscillatoria* spp. according to their growth phases, nutritional composition, biochemical (fatty acid and amino acid) composition, and pigment contents (chlorophyll, phycobiliprotein, carotenoid) to leverage their potential in diverse commercial applications.

## 2 Materials and Methods

### 2.1 Collection and Stock Culture of Pure Oscillatoria spp.

Two freshwater and two marine *Oscillatoria* spp. were isolated and morphologically identified according to their colony characteristics through microscopic observation (40 × magnification) (Nayeem, Dey, Dey, Karim, et al. 2024). The pure *Oscillatoria* isolates were procured from the Live Feed Research Corner of the Department of Aquaculture, Chattogram Veterinary and Animal Sciences University, Chattogram, Bangladesh. Marine *Oscillatoria* spp. comprised *Oscillatoria* sp. 1 (planktonic) and *Oscillatoria* sp. 2 (highly filamentous), while the freshwater species included *Oscillatoria* sp. 3 (moderately filamentous) and *Oscillatoria* sp. 4 (highly filamentous). Pure freshwater and marine stocks (100 mL) were inoculated in the 900 mL Bold's Basal Medium (BBM) (Bischoff and Bold, 1963) and Conway medium (Tompkins et al. 1995) respectively. The cultures were maintained at 24°C under continuous indoor light conditions of 12 h light: 12 h dark regime, with 2000 lx of light intensity (Bleakley and Hayes 2017).

### 2.2 Growth Curve Determination

*Oscillatoria* species were cultured in triplicate using clean and sterile 500 mL borosilicate Erlenmeyer flasks. Each flask contained 350 mL of liquid culture medium and was inoculated with 2%–3% pure culture stocks. The cultures were maintained at a temperature of 24°C ± 1°C with continuous 24‐h artificial light at an intensity of 150 μEm^−2^ s^−1^ and gentle aeration at 4.53 ± 0.53 mg/L. Cultures were continued until the death phase of *Oscillatoria* spp. The growth curve was determined based on optical density (absorbance) and chlorophyll content analysis.

#### 2.2.1 Determination of Chlorophyll‐a

Chlorophyll‐a from *Oscillatoria* sp. was extracted using a chemical method (Dixit et al. 2020). The algae samples (1 mL) were filtered, followed by 1 mL MgCO_3_ through filter paper, then folded to place in a 15 mL centrifuge tube containing acetone. After grinding with 10 mL of 90% acetone, the mixture was refrigerated for 1 h and centrifuged at 3000 rpm for around 10 min. The acetone extract was transferred to a new tube and then centrifuged at 500 rpm for 5 min, and its absorbance was measured against a blank (90% acetone).

Chlorophyll‐a concentration was determined using the spectrophotometric method (Jenkins 1982). The clear acetone extract was placed in a 1 cm cuvette, and optical density (OD) was measured at 750, 664, 647, and 630 nm wavelengths. The turbidity correction factor (OD value of 750 nm) was subtracted from the OD values at 664, 647, and 630 nm before their incorporation into the equations. The concentration of chlorophyll‐a was calculated using the adjusted OD values within the specified equations (Jeffrey and Humphrey 1975):

C_a_ = 11.85 (OD _664_) ‐ 1.54 (OD _647_) ‐ 0.08 (OD _630_)

Here, C_a_ is the chlorophyll‐a concentration in mg/L and OD values are the adjusted optical densities at the respective wavelengths. The pigment concentration per unit volume was assessed using the following formula:

$$\text{Chlorophyll}\;a\;\left(\mathrm{mg}/m3\right)=\frac{\mathrm{Ca}\;\left(\mathrm{mg}/L\;\right)\times \text{extract volume}\;\left(L\right)}{\text{volume of sample}\;\left(m^{3}\right)\;}$$

#### 2.2.2 Determination of Optical Density (OD)

The growth curve was analyzed by measuring optical density (OD) daily with a spectrophotometer (NanoDrop Spectrophotometer, Model‐Nanoplus, Germany), using culture media as the blank. Peak absorbance values of *Oscillatoria* spp. were recorded. Reference wavelengths of each culture were determined based on maximum absorbance peaks observed during spectral scanning of the culture samples between 300 and 700 nm. Species 1 and species 2 showed peak absorbance at 600 and 443 nm, respectively, while species 3 and species 4 exhibited the highest absorbance at 530 and 475 nm, respectively.

### 2.3 Mass Culture and Biomass Preparation

The mass culture of *Oscillatoria* spp. was conducted in plastic tanks using both BBM (Bischoff and Bold, 1963) and Conway medium (Tompkins et al. 1995). The stock cultures were then incrementally scaled up to 5, 8, 12, and finally 16 L in a 20 L plastic tank. PVC pipe substrates were used for filamentous *Oscillatoria* sp. culture to maximize the production. After reaching their early stationary phase, wet biomass was harvested through centrifugation at 5500 rpm for 5 min by using a centrifuge machine (TL5R Free Standing low‐speed refrigerated centrifuge, Herexi). The wet biomass was oven‐dried overnight at 40°C using a hot air oven (JSR Korea's Natural Convention Oven LNO‐150). A mortar and pestle were used to pulverize the dried biomass and then sieved to make fine powder. Then the dried powder was stored in a freezer at 4°C.

### 2.4 Nutritional Composition Determination

The protein content of the *Oscillatoria* samples was determined using the chemical method (Lowry et al. 1951). Freeze‐dried biomass (5 mg) was homogenized in 25 mL distilled water. A 0.5 mL aliquot was mixed with 0.5 mL of 1 N NaOH and heated at 100°C for 5 min. After cooling (10 min), 2.5 mL of a reagent mix comprising 50 mL Reactive 2 (2 g Na_2_CO_3_ in 100 mL 0.1 N NaOH) and 1 mL Reactive 1 (1% NP tartrate) was added. Following proper mixing, 0.5 mL of Folin reagent was added. The mixture was incubated in the dark (30 min). Spectrophotometric absorbance was measured at 750 nm. Protein content was calculated using a calibration curve from albumin standards (20–200 μg/L).

The lipid content was determined using the method described by Bligh and Dyer (1959) and Folch et al. (1957) methods. Each aluminum dish was labeled and initially weighed. An adequate sample (50 mg) was diluted with distilled water (5 × volume) in a centrifuge tube. Then, methanol: chloroform solution (2:1, v/v) was added, homogenized, and centrifuged at 1000 rpm for 4 min at 4°C. The supernatant was collected and kept on ice. The remaining pellet was re‐extracted with methanol: chloroform, centrifuged, and combined with the first supernatant. The combined supernatant was mixed with 1.5 mL of 0.9% NaCl, refrigerated for 1 h at 4°C, and centrifuged, forming two layers. The upper layer was discarded, and the lower layer was transferred to aluminum dishes, evaporated at 60°C, and weighed to determine the lipid content by subtracting the initial weight from the final weight.

The carbohydrate content was assessed according to the method of Dubois et al. (1956). Freeze‐dried biomass (5 mg) was homogenized in 25 mL distilled water, and 1 mL of the solution was mixed with 1 mL of 5% phenol and 5 mL of concentrated sulfuric acid. After a brief reaction (30 s) and cooling, the absorbance was measured at 488 nm. A glucose standard curve (20–140 μg/L) was used to calculate the carbohydrate content from the absorbance readings.

### 2.5 Analysis of Biochemical Composition

#### 2.5.1 Fatty Acid Determination

The fatty acid composition was analyzed using a slightly modified two‐step transesterification (2TE) method (Griffiths et al. 2010). *Oscillatoria* sp. powder (500 mg) was dissolved in 70 mL of diethyl ether for lipid extraction with a Digital Soxhlet Apparatus (FOOD ALYTRD40). The diethyl ether was evaporated at 60°C through a hot air oven. Subsequently, 1.5 mL methanolic NaOH was added to the lipid extract and sonicated at 80°C for 5 mi ns. After cooling to 25°C, 2 mL BF_3_ methanol was added, and the mixture was sonicated again at 80°C for 30 min. Following a second cooling (25°C), 1 mL isooctane and 5 mL saturated NaCl were added, well shaken to observe two layers. The upper layer containing fatty acid methyl esters (FAMEs) was transferred to a new test tube. A minute sample (1 mL) was taken for GC–MS analysis (GC‐2020plus, SHIMADZU, Japan), using a capillary column (30 m × 0.25 mm × 0.15 μm) with helium as the carrier gas (1.42 mL/min). The column temperature was programmed from 180°C to 280°C at 5°C/min and then at 280°C. Detection was performed by comparing the retention times of the FAMEs with those of a standard FAME mix (C8‐C24; Sigma‐Aldrich, Germany).

#### 2.5.2 Amino Acid Determination

The Moore and Stein method was slightly modified to identify amino acids (Moore and Stein 1951). The dried *Oscillatoria* sp. sample (1 g) was hydrolyzed for 24 h at 110°C ± 2°C in 25 mL of acidic solution (6 M HCl with 0.1% phenol). After cooling, samples were stabilized with Sample Dilution Buffer (SDB/Na) and the pH adjusted to 2.1–2.3. The hydrolysates were filtered, diluted with SDB/Na, and analyzed using a SYKAM S 433 amino acid analyzer with a UV detector. Nitrogen gas was used as the carrier gas (0.5 mL/min) at 60°C and a reproducibility of 3%. Amino acid concentrations were measured with Sigma‐Aldrich AA‐S‐18 standard and reported in mg/g, then converted to percentage of total amino acids.

### 2.6 Phycobiliproteins Determination

The spectrophotometric method was used to estimate the phycobiliproteins in *Oscillatoria* sp. (Siegelman and Kycia 1978). Dried cyanobacteria powder (40 mg) was mixed with 10 mL of 0.1 M phosphate buffer (pH 7.0) using a vortex mixer and stored at 4°C for 24 h. The samples were then centrifuged at 6000 rpm for 10 min. Absorbances of the sample extracts were measured at 562, 615, 652, and 720 nm using a spectrophotometer (NanoDrop Spectrophotometer, Model‐Nanoplus, Germany), with phosphate buffer as the blank. The phycocyanin (PC), allophycocyanin (APC) (Bennett and Bogorad 1973) and phycoerythrin (PE) (Siegelman and Kycia 1978) concentrations in the sample were calculated using the following formula:

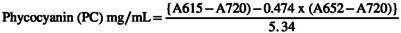

$$\text{Allophycocyanin}\;\left(\mathrm{APC}\right)\;\mathrm{mg}/\mathrm{mL}=\frac{\left\{A652-A720\right)-0.208\;x\;\left(A615-A720\right)\}}{5.09}$$

$$\text{Phycoerythrin}\;\left(\mathrm{PE}\right)\;\mathrm{mg}/\mathrm{mL}=\frac{\left\{A562-\left(2.41\;x\;\mathrm{PC}\right)-\left(0.849\;x\;\mathrm{APC}\right)\right\}}{9.62}$$

Total phycobiliproteins (mg/g) were calculated according to (Silveira et al. 2007) as follows:

$$P=\frac{\left(\text{Pigment concentration}\times V\right)}{\mathrm{DB}}$$

Where, V = volume of solvent, DB = dried biomass.

### 2.7 Purification Factor

The purity ratio, or purification factor, reflects the extent of impurities in the extracted pigment compound. The purity ratio of phycocyanin, phycoerythrin, and allophycocyanin extract was determined spectrophotometrically using A 620/A 280, A 565/A 280, and A 650/A 280 ratios (Bennett and Bogorad 1973).

### 2.8 Determination of Carotenoids

Carotenoid content was determined from the wet cyanobacterial suspension using the spectrophotometric method (Khatoon et al. 2020). The absorbance of the carotenoid extract was determined at a 450 nm wavelength through a spectrophotometer. The carotenoid content in the samples, expressed in micrograms, was calculated by multiplying 25.2 by the absorbance of 450 nm (A_450_) (Shaish et al. 1992).

### 2.9 Statistical Analysis

Statistical analyses regarding the optical density, protein, lipid, carbohydrate content, amino acid, fatty acid, and pigment contents were performed using the software IBM SPSS (v. 26.0) and R (version 4.4.1) in RStudio (version 2024.04.2 + 764). Data visualization was conducted using R in RStudio utilizing packages such as “ggplot2” for plotting and “dplyr” for data manipulation. Descriptive statistics for each parameter of *Oscillatoria* sp. were performed. Homogeneity of variance test was also performed. The collected data were analyzed using a one‐way analysis of variance (ANOVA), and significant variations among *Oscillatoria* species were determined through Tukey's multiple comparison test at a 95% confidence level. A post hoc test was conducted to identify differences between groups.

## 3 Results

### 3.1 Growth Curve Determination of Oscillatoria spp.

**FIGURE 1:**
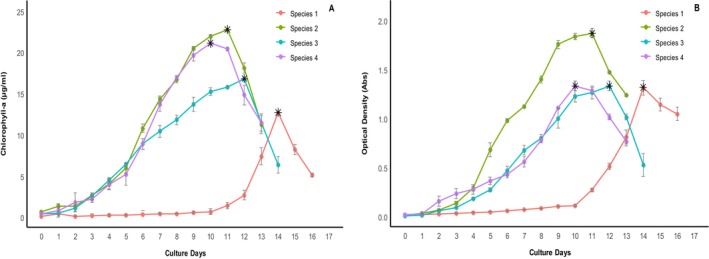
Growth curve of *Oscillatoria* spp. in terms of chlorophyll‐a content (μg/mL) (A) and optical density (B). Values are averages of the triplicates with standard error.

The growth curve of each *Oscillatoria* sp. was obtained based on chlorophyll‐a content and optical density as a function of culture duration (Figure 1). Growth phases of each *Oscillatoria* sp. were observed to vary significantly (*p* < 0.05). *Oscillatoria* sp. 2 exhibited significantly higher (*p* < 0.05) chlorophyll‐a (22.72 ± 0.04 μg/mL) and OD value (1.87 ± 0.03) on the 11th day compared to other species. *Oscillatoria* sp. 4 attains an exponential phase in a shorter period (on the 10th day) while *Oscillatoria* sp. 1 attains maturity on the 14th day (Nayeem, Dey, Dey, Karim, et al. 2024).

### 3.2 Biomass

**FIGURE 2:**
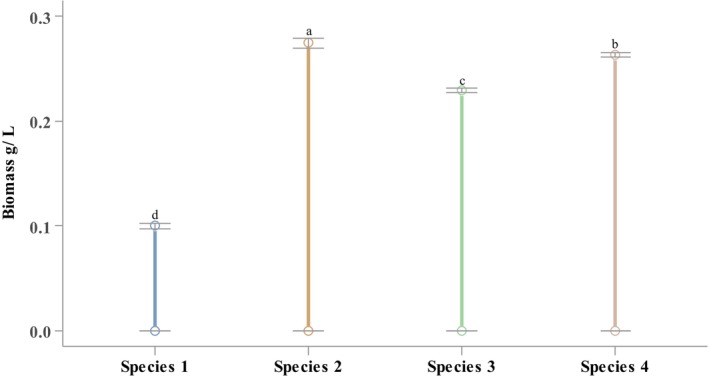
Dried biomass obtained from *Oscillatoria* spp. Values are the mean of the triplicates with standard error bars. Values in each category with a different letter indicate significant variations among the species (*p* < 0.05).

The dried biomass of *Oscillatoria* sp. was varied from 0.10 ± 0.01 to 0.28 ± 0.01 g/L. Significantly (*p* < 0.05; η^2^ = 0.996) higher and lower dried biomass was recorded from *Oscillatoria* sp. 2 and *Oscillatoria* sp. 1 respectively (Figure 2) (Nayeem, Dey, Dey, Karim, et al. 2024).

### 3.3 Nutritional Composition

**FIGURE 3:**
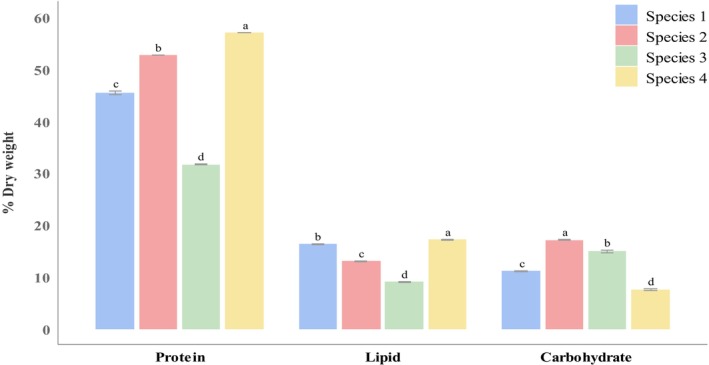
Nutritional composition of dried *Oscillatoria* spp. including protein, lipid, and carbohydrate content. Significant variations among the species (*p* < 0.05) are indicated by values (mean ± SE) in each series with a distinct letter.

Significant differences (*p* < 0.05) in the nutritional composition of *Oscillatoria* spp. are illustrated in Figure 3, highlighting the estimated percentages of crude protein, lipid, and carbohydrate content derived from the dried biomass. Filamentous *Oscillatoria* spp. exhibited higher protein and lipid, and lower carbohydrate levels than planktonic *Oscillatoria* sp. Significant (*p* < 0.05) higher protein (56.97% ± 0.03%) and lipid (17.13% ± 0.13%) were obtained from *Oscillatoria* sp. 4, whereas *Oscillatoria* sp. 2 showed higher carbohydrate (17.04% ± 0.08%) compared to other species (Nayeem, Dey, Dey, Karim, et al. 2024). These differences were statistically significant (*p* < 0.05) and supported by very large effect sizes: protein (η^2^ = 0.999), lipid (η^2^ = 0.998), and carbohydrate (η^2^ = 0.995), indicating strong group effects.

### 3.4 Biochemical Composition

#### 3.4.1 Fatty Acid Composition

**FIGURE 4:**
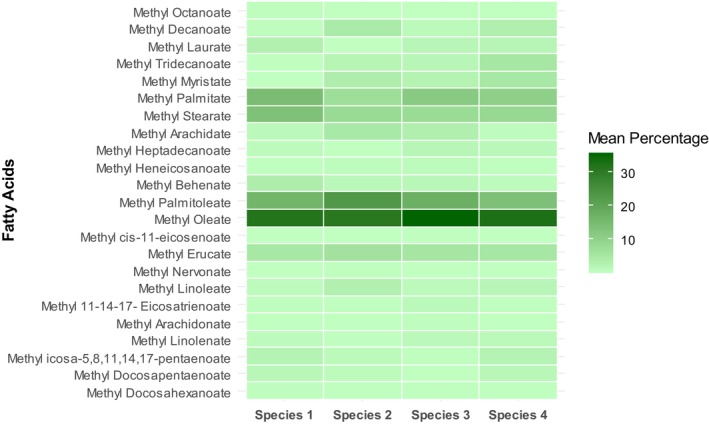
Heat map depicting percentage levels of fatty acid profiles in *Oscillatoria* spp., the color scale ranges from light green (low percentage) to dark green (high percentage), illustrating the variation in fatty acid abundance. Data are the mean of the duplicates.

Variations in the percentages of individual fatty acids from dried *Oscillatoria* spp. were measured and depicted in Figure 4 as a heat map. This heat map illustrates that methyl oleate (32.00% ± 0.17% to 36.03% ± 0.50%) and methyl palmitoleate (13.97% ± 0.05% to 23.17% ± 0.09%) were abundant in both freshwater and marine *Oscillatoria* sp. Table 1 exhibited the total percentages of saturated, monounsaturated, and polyunsaturated fatty acids in *Oscillatoria* spp., highlighting significant (*p* < 0.05) differences and effect sizes (η_SAFA_^2^ = 0.951, η_MUFA_^2^ = 0.996, η_PUFA_^2^ = 0.92) among these categories. Monounsaturated fatty acids were prevalent (52.36% ± 0.37%–60.96% ± 0.31%) in *Oscillatoria* spp. followed by saturated fatty acids (33.27% ± 0.33%–39.71% ± 0.89%) and polyunsaturated fatty acids (4.49% ± 0.01%–8.15% ± 0.07%) (Nayeem, Dey, Dey, Karim, et al. 2024).

**TABLE 1:** Fatty acid contents (% total) of *Oscillatoria* spp. Each value is the mean of the duplicates with standard error.

Fatty acids	*Oscillatoria*			
	Species 1	Species 2	Species 3	Species 4
SAFA	39.71 ± 0.89^a^	33.27 ± 0.33^d^	35.69 ± 0.37^c^	38.55 ± 0.52^b^
MUFA	53.57 ± 0.17^c^	60.96 ± 0.31^a^	59.55 ± 0.02^b^	52.36 ± 0.37^d^
PUFA	6.72 ± 0.28^b^	5.76 ± 0.12^c^	4.49 ± 0.01^d^	8.15 ± 0.07^a^
n6‐PUFA	1.25 ± 0.07^d^	3.45 ± 0.12^a^	2.85 ± 0.02^b^	2.37 ± 0.07^c^
n3‐PUFA	5.47 ± 0.35^b^	2.31 ± 0.00^c^	1.64 ± 0.03^d^	5.78 ± 0.00^a^

*Note:* Significant (*p* < 0.05) Oscillatoria spp. variations are denoted by values in each series with a distinct letter. Data are the means of the duplicates.

Abberevations: MUFA, Monounsaturated fatty acids; n3‐PUFA, Omega‐3 PUFA; n6‐PUFA, Omega‐6 PUFA; PUFA, Polyunsaturated fatty acids; SAFA, Saturated fatty acids.

#### 3.4.2 Amino Acid Composition

**FIGURE 5:**
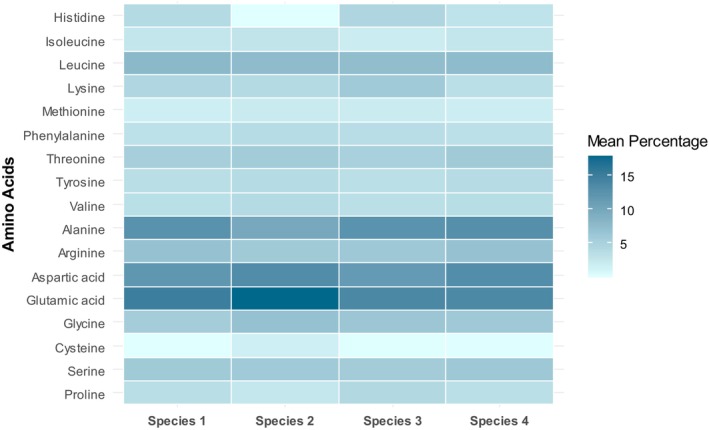
Heat map depicting percentage levels of amino acid profiles in *Oscillatoria* spp. Values are the mean of the duplicates. The color scale ranges from light blue (low percentage) to dark blue (high percentage), illustrating the variation in amino acid abundance.

The individual amino acids (%) in dried *Oscillatoria* spp. were analyzed and displayed as a heat map in Figure 5. *Oscillatoria* spp. were rich in non‐essential amino acids, including glutamic acid, aspartic acid, and alanine. *Oscillatoria* sp. 2 showed the highest glutamic acid (17.93% ± 0.05%) and aspartic acid (13.33% ± 0.04%) while *Oscillatoria* sp. 4 exhibited the highest alanine (12.99% ± 0.05%) content. Total percentages of essential and non‐essential amino acids in *Oscillatoria* spp. with significant (*p* < 0.05), (η_EAA_^2^ = 0.997, η_NEAA_^2^ = 0.996) differences among the species were demonstrated in Table 2. Non‐essential amino acids about 61.12% ± 0.05% to 64.62% ± 0.03% were found to be the most abundant compared to essential amino acids about 35.38% ± 0.02% to 38.84% ± 0.04% (Nayeem, Dey, Dey, Karim, et al. 2024).

**TABLE 2:** Amino acid content (% total) of *Oscillatoria* spp.

*Oscillatoria*	Essential amino acid (EAA)	Non‐essential amino acid (NEAA)
Species 1	37.58 ± 0.06^b^	62.37 ± 0.03^c^
Species 2	35.38 ± 0.02^d^	64.62 ± 0.03^a^
Species 3	38.84 ± 0.04^a^	61.12 ± 0.05^d^
Species 4	36.72 ± 0.12^c^	63.23 ± 0.15^b^

*Note:* Values are average of the duplicates (mean ± SE). Significant (*p* < 0.05) Oscillatoria spp. variations are denoted by values in each series with a distinct letter.

### 3.5 Pigments

*Oscillatoria* spp. exhibited significant (*p* < 0.05) variations in pigment contents. Chlorophyll‐a, carotenoid, and phycobiliprotein contents were assessed and presented in Table 3. Significantly, both chlorophyll‐a (*p* < 0.05; η^2^ = 0.997) and carotenoid contents (*p* < 0.05; η^2^ = 0.994) were found to be highest in *Oscillatoria* sp. 2, while the lowest levels were observed in *Oscillatoria* sp. 1. *Oscillatoria* sp. 4 exhibited the highest phycobiliprotein content (η^2^ = 0.9996) compared to others.

**TABLE 3:** Pigment contents of *Oscillatoria* spp. Values are the mean of the triplicates with standard error (SE = σ/√n).

Pigment contents	*Oscillatoria*			
	Species 1	Species 2	Species 3	Species 4
Chlorophyll‐a (μg/ml)	12.67 ± 0.04^d^	22.72 ± 0.04^a^	16.76 ± 0.04^c^	21.06 ± 0.07^b^
Carotenoid (μg/ml)	1.00 ± 0.01^d^	1.40 ± 0.01^a^	1.05 ± 0.01^c^	1.20 ± 0.01^b^
Phycocyanin (mg/g)	81.85 ± 0.84^c^	84.75 ± 0.12^b^	69.00 ± 0.12^d^	93.47 ± 0.08^a^
Allophycocyanin (mg/g)	10.78 ± 1.00^b^	18.19 ± 0.06^a^	17.00 ± 0.12^a^	16.13 ± 0.12^a^
Phycoerythrin (mg/g)	0.36 ± 0.14^d^	8.10 ± 0.15^b^	1.40 ± 0.21^c^	11.82 ± 0.14^a^
Total phycobiliproteins (mg/g)	93.00 ± 0.23^c^	111.04 ± 0.06^b^	87.39 ± 0.12^d^	121.42 ± 0.06^a^

*Note:* Significant differences (*p* < 0.05) are marked by unique letters for each value in the series (Nayeem, Dey, Dey, Debi, et al. 2024).

**FIGURE 6:**
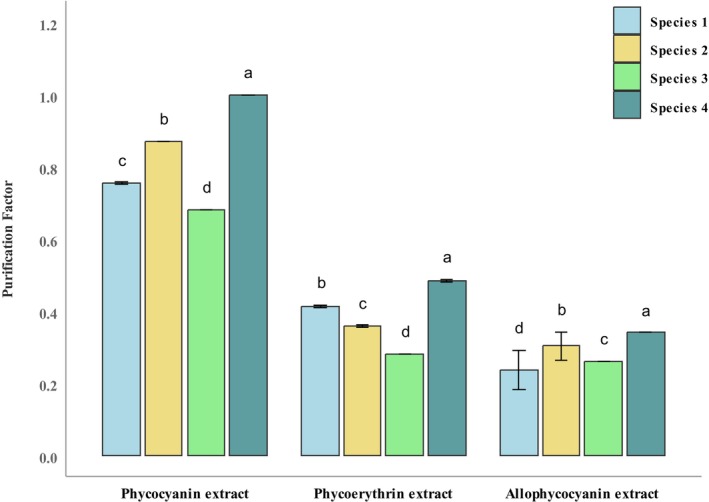
Purification factors for raw extracts of phycocyanin, phycoerythrin, and allophycocyanin in *Oscillatoria* spp. Significant variations among the *Oscillatoria* spp. (*p* < 0.05) are indicated by values with a distinct letter in each category.

The purification factors for phycocyanin, phycoerythrin, and allophycocyanin in raw extracts of *Oscillatoria* sp. are illustrated in Figure 6. The highest values for phycocyanin, allophycocyanin, and phycoerythrin were observed in *Oscillatoria* sp. 4 (1.00 ± 0.00, 0.34 ± 0.00, 0.48 ± 0.00), while the lowest were found in *Oscillatoria* sp. 3 (0.68 ± 0.00, 0.26 ± 0.00, 0.28 ± 0.00). The significant differences (*p* < 0.05) for phycocyanin (η^2^ = 0.999) and phycoerythrin (η^2^ = 0.9999) were observed. No significant differences (*p* > 0.05; η^2^ = 0.748) were observed in the purity of allophycocyanin raw extracts among the species (*p* = 0.188) (Nayeem, Dey, Dey, Debi, et al. 2024).

## 4 Discussion

In the quest for sustainable approaches and novel applications, *Oscillatoria* stands out as a promising cyanobacterial candidate due to its diverse physiological and biochemical properties. However, to fully harness its potential, a thorough characterization was essential. Analyzing growth curves, nutritional compositions, biochemical profiles, and pigment contents addressed the need for optimized species screening and selection for advancements in sustainable technologies and industrial applications.

### 4.1 Growth Curve Determination of Oscillatoria spp.

*Oscillatoria* spp. were harvested in their early stationary phase, making it essential to analyze the growth curve to identify the point of stationary phase with the highest productivity. In this research, the growth curves of *Oscillatoria* spp. exhibited differential maturity levels. Peak maturity of *Oscillatoria* species 1, species 2, species 3, and species 4 was attained at days 14, 11, 12, and 10. Notably, *Oscillatoria* species 2 and species 4 exhibited maximum chlorophyll‐a over a shorter duration with the exponential phase of species 2 between days 5 and 111 and species 4 between days 5 and 100. Planktonic *Oscillatoria* sp. exhibited a longer growth curve with extended lag and log phases due to their adaptation to more variable and nutrient‐rich environments. In contrast, filamentous *Oscillatoria* spp. showed faster growth due to their efficient nutrient uptake and optimized structure for resource acquisition. *Oscillatoria* spp. may exhibit differential growth patterns and maturation times due to variations in the species, filamentous degree, experimental conditions, and culture techniques. So, each species needs specific optimization for commercial use. A freshwater *Oscillatoria* sp. reached maturity by day 8 of a 9‐day culture period with peak chlorophyll‐a concentrations (Kasan et al. 2015) which was nearly consistent with our findings of *Oscillatoria* sp. 4, while another freshwater *Oscillatoria subbrevis* matured later, between days 18 and 20 (Sarmah and Rout 2018). Thus, highly filamentous *Oscillatoria* species 2 and species 4 can be cultivated commercially to obtain higher productivity of chlorophyll‐a and biomass within a short period.

### 4.2 Biomass

Filamentous *Oscillatoria* spp. exhibited higher dried biomass yields compared to planktonic forms. Marine *Oscillatoria* sp. 2 yielded the highest biomass at 0.28 ± 0.01 gL^−1^, and marine planktonic *Oscillatoria* sp. 1 showed the lowest biomass at 0.10 ± 0.01 gL^−1^. In a study, *Oscillatoria sancta* accumulated maximum dry‐weight biomass of 0.552 ± 0.002 gL^−1^ (Touliabah and Refaay 2023). *Oscillatoria* sp. BTA‐170 was cultured in a photobioreactor over 15 days, resulting in a biomass of 3.1 g/L (Sharma et al. 2022). In a raceway reactor, *Oscillatoria* sp. biomass concentration varied between 0.3 and 0.9 g/L (Morillas‐España et al. 2022). Biomass variation of *Oscillatoria* spp. may be driven by differences in reactor, physicochemical conditions, species‐specific attributes, filamentous degree, cultivation protocols, harvesting, and drying methods. Factors like light intensity, nutrient concentrations, and temperature significantly influence biomass accumulation.

### 4.3 Nutritional Compositions

Nutritional composition of *Oscillatoria* spp. including crude protein (31.56% ± 0.09% to 56.97% ± 0.03%) and crude lipid (9.07% ± 0.07% to 17.13% ± 0.13%) were found higher in freshwater *Oscillatoria* sp. 4, and crude carbohydrate (7.49% ± 0.15% to 17.04% ± 0.08%) was found higher in marine planktonic *Oscillatoria* sp. 1. Nutritional composition differences in the freshwater and marine *Oscillatoria* sp. are primarily driven by differences in nutrient availability, salinity, water quality, culture, and drying conditions, which influence metabolic processes and biochemical composition. Higher protein and lipid content of filamentous *Oscillatoria* sp. can be due to its larger surface area and enhanced nutrient absorption, which supports robust growth and metabolic activities. Planktonic *Oscillatoria* sp. may rely more on carbohydrate reserves than filamentous *Oscillatoria* sp. for continuous energy, adaptation, growth, and survival. In *Microcystis aeruginosa* , intrinsic carbohydrate and extracellular polysaccharide (EPS) accumulation enhance buoyancy and colony formation, supporting planktonic survival in dynamic aquatic environments. These carbon allocation strategies function independently of stress, serving as stable metabolic buffers and ecological adaptations (Wei et al. 2020; Ofaim et al. 2021). As this study continued under non‐stressed conditions, the elevated carbohydrate of planktonic *Oscillatoria* sp. may be due to species‐specific metabolic and ecological adaptation. Earlier studies reported that dried cyanobacterial biomass contains higher quantities of protein (40%–60%), lipids (5%–15%) and carbohydrates (10%–30%) (Safi et al. 2013; Singh, Kumari, et al. 2016). The biomass of *Oscillatoria* sp. BTA 170 comprised 37.44% protein, 22.31% lipids, and 32.25% carbohydrates (Tiwari et al. 2019). Another freshwater *Oscillatoria* sp. comprised 57.98% ± 2.54% protein, 8.85% ± 2.32% lipid, and 26.59% ± 2.43% carbohydrate cultured in 25°C (Idenyi et al. 2021). The biomass of *Oscillatoria* sp. cultured in wastewater was also measured to have 32.9% ± 1.92% protein, 11.76% ± 0.79% lipids, and 27.36% ± 3.78% carbohydrates (Rasheedy et al. 2017). *Nostoc* sp. with the lipid content of 15.7% has been reported as the potential candidate to produce biodiesel (Nagappan et al. 2020). *Oscillatoria* sp. *FW01* was also found promising with rich lipid content (10.2%) and favorable fatty acid profile for biodiesel production compared to *Phormidium* sp. *FW01, Phormidium* sp. *FW02*, and *Oscillatoria* sp. *FW02*, which had lipid contents of 6.7%, 8.2%, and 9.4%, respectively (Yadav et al. 2021).

So, nutritional compositions are highly variable in *Oscillatoria* sp. Protein‐rich *Oscillatoria* spp. may boost metabolic efficiency, making them ideal for bioremediation and supplementation in animal feed. Carbohydrates can provide an energy source, supporting growth and resilience, and can be fermented into bioethanol. In *Oscillatoria* spp., neutral lipids, mainly triacylglycerols (TAGs) are the dominant lipid class, serving as key substrates for biodiesel production (Yadav et al. 2021). Lipids from *Oscillatoria* are converted into biofuel through transesterification, contributing to sustainable energy production, while byproducts like glycerol can be utilized in various industrial processes (Farouk et al. 2024).

### 4.4 Biochemical Composition

#### 4.4.1 Fatty Acid Composition

Fatty acids were found variable in *Oscillatoria* spp. and revealed significant details about its lipid quality. Saturated and mono‐unsaturated fatty acids were prevalently present in marine planktonic *Oscillatoria* sp. 1 (39.71% ± 0.89%) and filamentous *Oscillatoria* sp. 2 (60.96% ± 0.31%) respectively. Polyunsaturated fatty acids were found higher in freshwater *Oscillatoria* sp. 4 (8.15% ± 0.07%). Monounsaturated fatty acids, including Methyl Palmitoleate (C16:1) and Methyl Oleate (C18:1) are prevalent in *Oscillatoria* spp. In contrast, saturated fatty acid, including methyl stearate (C18:0) was detected between 8.03% ± 0.40% and 13.85% ± 0.70%, and palmitic acid (C16:0) was found in the range of 7.34% ± 0.37% to 14.82% ± 0.18%. Differences in growth conditions (light and temperature), nutrient availability, species, and metabolic pathways of lipid influence the types and proportions of fatty acids produced. Key fatty acids that influence biofuel quality include palmitic acid (C16:0), stearic acid (C18:0), oleic acid (C18:1), linoleic acid (C18:2), and linolenic acid (C18:3) (Lee et al. 2011). Saturated fatty acids, specifically palmitic acid (C16:0) at 31.5% and stearic acid (C18:0) at 60.7%, were found to be higher in freshwater *Oscillatoria* sp. (Idenyi et al. 2021). *Oscillatoria* sp. also exhibited a fatty acid profile consisting of 34.74% saturated fatty acids (SFA), 60.63% monounsaturated fatty acids (MUFA), and 4.3% polyunsaturated fatty acids (PUFA) (Irmak and Arzu 2020) which closely aligned with our findings. Fatty acids from *Oscillatoria* sp. *FW01* contained desirable fatty acids (C16:0; C16:1; C18:1; C18:3) for producing high‐quality biodiesel (Yadav et al. 2021), which is also consistent with our findings.

Mundt et al. (2003) demonstrated that fatty acids inhibited the growth of gram‐positive bacteria such as *Micrococcus flavus* , *Staphylococcus aureus* , and *Bacillus subtilis* in agar diffusion tests. This suggests that the rich fatty acid profile in *Oscillatoria* species contributes to its antimicrobial properties. High levels of saturated fatty acids also provide excellent resistance to oxidation in biofuels of Spirulina platensis (Mostafa and El‐Gendy 2017). Additionally, monounsaturated fatty acids (MUFAs) are crucial for biodiesel production due to their favorable properties at low temperatures (Cao et al. 2014). A high proportion of SAFAs and MUFAs are also preferred for increasing energy yield and superior oxidative stability (Musharraf et al. 2012). Alkaline transesterification of *Oscillatoria annae* triglycerides resulted in a higher biodiesel yield (86% w/v) compared to the 76.5% w/v yield obtained using lipase‐catalyzed conversion (Vimalarasan et al. 2011). Their capacity to utilize external fatty acids further enhances their value in diverse industries such as biofuels, pharmaceuticals, materials, cosmetics, and agriculture (Kahn et al. 2023).

#### 4.4.2 Amino Acid Composition

Amino acids (AAs) in protein metabolism are divided into nonessential (e.g., aspartic acid, glutamic acid) and essential (e.g., valine, leucine) categories. Certain nonessential AAs, like cysteine and proline, become conditionally essential when their demand exceeds their rate of synthesis. The amino acid content in algal biomass is directly influenced by the cultivation methods and growth conditions, such as the chemical composition of the culture medium, light quality, pH, temperature, salinity, and turbulence (Kolmakova and Kolmakov 2019). Variable amino acids were recorded from *Oscillatoria* spp. in this research. Nonessential amino acids including glutamic acid (13.74% ± 0.05%–17.93% ± 0.05%), aspartic acid (11.63% ± 0.02%–13.33% ± 0.04%), and alanine (9.96% ± 0.04%–12.99% ± 0.05%) are prevalent in *Oscillatoria* spp. Leucine (7.51% ± 0.01%–7.98% ± 0.03%) and threonine (5.20% ± 0.03%–6.00% ± 0.04%) are predominant as essential amino acids in *Oscillatoria* spp. Proline ranged between 2.72% ± 0.03% to 4.34% ± 0.01% in *Oscillatoria* spp. However, in a recent study, conditional essential amino acids such as proline were reported to be predominant (94.3% ± 0.9%) in freshwater *Oscillatoria brevis* , while essential and nonessential amino acids only comprised 3.64% ± 0.1% and 1.1% ± 0.03%, respectively (Moniruzzaman et al. 2023). This discrepancy may arise from differences in species, isolation and purification processes, growth media, culture procedures, and drying methods, as the growth conditions and amino acid assessments in both studies varied significantly. The prevalent nonessential amino acids, while not classified as dietary essentials, play important roles in supporting gut health, nitrogen metabolism, and overall metabolic balance, making the biomass valuable as a supplementary protein source in feed or nutraceutical formulations (Tresia et al. 2023).

### 4.5 Pigments

*Oscillatoria* sp. is a great source of natural pigments, including chlorophylls, carotenoids, and characteristic phycobiliproteins (Morillas‐España et al. 2022). Pigment contents were assessed in *Oscillatoria* spp., and higher chlorophyll‐a content was recorded ranging from 12.67 ± 0.04 to 22.72 ± 0.04 μg/mL and carotenoid content from 1.00 ± 0.01 to 1.40 ± 0.01 μg/mL. Results of marine *Oscillatoria* sp. 2 were consistent with Zavřel et al. (2015), who reported similar values (22 μg/mL) for cyanobacterial chlorophyll. In contrast, Idenyi et al. (2021) observed lower chlorophyll‐a content (0.88 ± 0.08 μg/mL) and carotenoid content (0.41 ± 0.05 μg/mL), highlighting a discrepancy in the observed concentrations. Chlorophyll‐a variation among *Oscillatoria* species arises from differences in their light‐harvesting systems, including the structure and composition of core antenna complexes. These differences are driven by genetic factors, environmental adaptations, species and culture conditions, and the extraction process, which influence pigment synthesis and composition.

The World Health Organization has established recommended limits for cyanobacteria abundance and chlorophyll‐a concentration, classifying them into low (< 10 g/L), moderate (10 to 50 g/L), high (50 to 5000 g/L), and extremely high risk (> 5000 g/L) categories (Chorus and Welker 2021). *Oscillatoria* spp. showed low blooming potential (< 10 g/L). Therefore, the high chlorophyll‐a content combined with low blooming potential in *Oscillatoria* sp. suggests promising prospects for high productivity and biomass production. Carotenoids also offer protection against oxidative damage and enumerate the antioxidant potential of *Oscillatoria* spp.

Phycobiliproteins are bright, fluorescent pigments found in cyanobacteria and some algae, providing blue‐green colors. (Morillas‐España et al. 2022). Phycobiliproteins, including phycocyanin, allophycocyanin, and phycoerythrin, were extracted and measured from *Oscillatoria* spp. in this research. A higher amount of phycobiliprotein content was recorded from both highly filamentous freshwater and marine *Oscillatoria* spp. Total phycobiliprotein content was found to be 87.39 ± 0.12 to 121.42 ± 0.06 mg/g, where prevalently phycocyanin (C‐PC) content (mg/g) ranged from 69.00 ± 0.12 to 93.47 ± 0.08. Allophycocyanin content (APC) ranged from 10.78 ± 1.00 to 18.19 ± 0.06 mg/g, and phycoerythrin content (PE) ranged from 0.36 ± 0.14 to 11.82 ± 0.14 mg/g. Maximum purity of phycocyanin, phycoerythrin, and allophycocyanin was about 1.00 ± 0.00, 0.48 ± 0.00, and 0.34 ± 0.00, respectively. In earlier studies, C‐phycocyanin concentration of marine *Oscillatoria* sp. was reported between 22 and 106 mg/L, yielding colors comparable to those found in commercial products. The final extract purity ratio of 0.71 makes the extract suitable for direct use as a food colorant (Morillas‐España et al. 2022). Under optimized conditions, the concentrations of phycobiliproteins (PBPs) were notably higher than in the control, with values of 15.21%, 3.95%, and 1.89% (w/w) for C‐PC, APC, and PE, respectively. The purity of C‐PC, APC, and PE under optimized conditions was found to be 0.87, 0.38, and 0.6, respectively (Zuorro et al. 2021). From freshwater *Oscillatoria* sp., 8.7% C‐PC (w/w), 3.8% APC (w/w) and 4.1% PE (w/w) were extracted (Contreras‐Ropero et al. 2022) which is much lower than our findings.

The commercial value of phycobiliprotein hinges on its purity grade; phycobiliprotein purity levels above 0.7 are classified as food grade, over 1.5 as cosmetic grade, exceeding 3.9 as reactive grade, and above 4.0 as analytical grade (Sala et al. 2018; Patil and Raghavarao 2007). Phycocyanin derived from *Oscillatoria minima* demonstrates antimicrobial, algicidal, and antiradical effects, as confirmed through both in silico and in vitro evaluations (Venugopal et al. 2020). A recent industrial biorefinery approach for *Arthrospira platensis* produced crude phycocyanin extract with purity around 0.54 (food‐grade entry level). Subsequent downstream processing (dialysis + precipitation) enhanced the purity to around 3.5, making it suitable for analytical grade use in nutraceutical and pharmaceutical industries (Sánchez‐Laso et al. 2023). Phycobiliproteins from *Oscillatoria* spp. in this research can be classified as food‐grade natural pigments, making them suitable for commercialization in various industries as colorants, fluorescent markers, nutraceuticals, and pharmaceuticals.

## 5 Conclusions

This comprehensive analysis of freshwater and marine *Oscillatoria* spp. reveals substantial variability in growth metrics, nutritional composition, and biochemical profiles. Highly filamentous species exhibited higher protein and lipid levels. The prevalence of unsaturated fatty acids and non‐essential amino acids highlights their potential for biofuel production and as a supplementary protein source in animal feed. Significant levels of phycobiliproteins, notably phycocyanin, can be used as natural coloring agents and have potential applications in the dyeing industry. These proteins offer valuable applications ranging from fluorescent markers to therapeutic agents. The findings support the strategic selection and optimization of *Oscillatoria* spp. for commercial use, advancing both sustainable energy solutions and therapeutic innovations. Further research should explore the secondary bioactive compounds in *Oscillatoria* spp., as well as their potential for bioenergy production and bioremediation. Enhanced cultivation practices will be essential for unlocking their full commercial and therapeutic potential, including antimicrobial and anti‐cancer properties. These efforts could pave the way for significant innovations and developments across multiple fields.

## Author Contributions

**Jannatul Nayeem:** data curation (lead), formal analysis (lead), methodology (lead), writing – original draft (lead). **Proma Dey:** data curation (equal), formal analysis (equal). **Sumit Kanti Dey:** methodology (equal), writing – original draft (equal). **Helena Khatoon:** conceptualization (equal), project administration (lead), resources (lead), supervision (lead), writing – review and editing (equal).

## Conflicts of Interest

The authors declare no conflicts of interest.

## Supporting information


**Table S1:** Variability in fatty acids (%) among different *Oscillatoria* spp. (mean ± SE).

## Data Availability

https://data.mendeley.com/datasets/v9xwnvn39p/1, https://data.mendeley.com/datasets/tchp9jznkb/1.
