# Supplementary material for: Unveiling the Biological Potential of Indigenous Oscillatoria spp. From Freshwater and Marine Ecosystems Through Advanced Characterization
Source: Food Sci Nutr. 2025 Sep 1;13(9):e70868. doi: 10.1002/fsn3.70868 (PMC12401714; doi:10.1002/fsn3.70868)
Supplement: Supplementary file 1 — Table S1: Variability in fatty acids (%) among different Oscillatoria spp. (mean ± SE). [file FSN3-13-e70868-s001.docx]

**Supplementary Table 1.** Variability in fatty acids (%) among different *Oscillatoria* spp. (mean± SE).

| **Carbon** | **Fatty Acid Methyl Esters** | ***Oscillatoria*** | | | | |
| --- | --- | --- | --- | --- | --- | --- |
|  |  | **Species 1** | **Species 2** | **Species 3** | **Species 4** | |
|  |  | **Amount (%)** | | | | |
| **Saturated Fatty Acid (SAFA)** | | | | | | |
| C8:0 | Methyl Octanoate | 0.48±0.05 | 0.05±0.00 | 0.03±0.00 | | 0.02±0.00 |
| C10:0 | Methyl Decanoate | 0.46±0.01 | 4.48±0.20 | 1.16±0.15 | | 3.57±0.02 |
| C12:0 | Methyl Laurate | 3.30±0.06 | 0.04±0.00 | 2.02±0.00 | | 2.23±0.00 |
| C13:0 | Methyl Tridecanoate | 0.27±0.01 | 2.30±0.09 | 1.89±0.03 | | 5.31±0.00 |
| C14:0 | Methyl Myristate | 0.06±0.00 | 3.97±0.32 | 2.74±0.22 | | 5.18±0.49 |
| C16:0 | Methyl Palmitate | 14.82±0.18 | 7.34±0.37 | 11.83±0.46 | | 10.37±0.14 |
| C18:0 | Methyl Stearate | 13.85±0.70 | 8.14±0.03 | 8.03±0.40 | | 8.59±0.04 |
| C20:0 | Methyl Arachidate | 1.36±0.09 | 4.84±0.02 | 3.49±0.36 | | 0.59±0.00 |
| C17:0 | Methyl Heptadecanoate | 0.97±0.00 | 0.04±0.00 | 1.92±0.98 | | 1.55±0.04 |
| C21:0 | Methyl Heneicosanoate | 0.22±0.02 | 0.57±0.09 | 0.82±0.12 | | 0.27±0.02 |
| C22:0 | Methyl Behenate | 3.91±0.05 | 1.51±0.04 | 1.76±0.06 | | 0.89±0.01 |
| C23:0 | Methyl Tricosanoate | ND±ND | ND±ND | ND±ND | | ND±ND |
| C24:0 | Methyl Lignocerate | ND±ND | ND±ND | ND±ND | | ND±ND |
| **Mono Unsaturated Fatty Acid (MUFA)** | | | | | | |
| C16:1 | Methyl Palmitoleate | 16.43±0.03 | 23.17±0.09 | 17.74±0.01 | | 13.97±0.05 |
| C18:1 | Methyl Oleate | 32.00±0.17 | 31.07±0.09 | 36.03±0.50 | | 32.96±0.45 |
| C20:1 | Methyl cis-11-eicosenoate | 0.01±0.01 | 0.04±0.02 | 0.17±0.01 | | 0.03±0.01 |
| C22:1 | Methyl Erucate | 5.13±0.02 | 6.47±0.18 | 5.52±0.52 | | 5.35±0.02 |
| C24:1 | Methyl Nervonate | 0.00±0.00 | 0.21±0.11 | 0.09±0.04 | | 0.05±0.01 |
| **Poly Unsaturated Fatty Acid (PUFA)** | | | | | | |
| C18:2n-6 | Methyl Linoleate | 0.77±0.05 | 3.25±0.10 | 1.16±0.06 | | 2.05±0.01 |
| C20:3n-6 | Methyl 11-14-17- Eicosatrienoate | 0.44±0.03 | 0.15±0.06 | 1.22±0.05 | | 0.22±0.09 |
| C20:4n-6 | Methyl Arachidonate | 0.04±0.01 | 0.05±0.03 | 0.47±0.00 | | 0.10±0.03 |
| C18:3n-3 | Methyl Linolenate | 0.89±0.03 | 0.25±0.00 | 1.22±0.01 | | 1.23±0.00 |
| C20:5n-3 | Methyl icosa-5,8,11, 14,17-pentaenoate | 2.58±0.05 | 1.13±0.00 | 0.27±0.04 | | 2.60±0.05 |
| C22:5n-3 | Methyl Docosapentaenoate | 1.67±0.47 | 0.80±0.00 | 0.01±0.00 | | 1.75±0.05 |
| C22:6n-3 | Methyl Docosahexanoate | 0.33±0.04 | 0.13±0.00 | 0.15±0.00 | | 0.20±0.00 |
